# Early relapse rate determines further relapse risk: results of a 5-year follow-up study on pediatric CFH-Ab HUS

**DOI:** 10.1007/s00467-020-04751-9

**Published:** 2020-10-07

**Authors:** Johannes Hofer, Magdalena Riedl Khursigara, Markus Perl, Thomas Giner, Alejandra Rosales, Gerard Cortina, Siegfied Waldegger, Therese Jungraithmayr, Reinhard Würzner

**Affiliations:** 1 Institute of Neurology of Senses and Language, Hospital of St John of God, Linz, Austria; 2 Research Institute of Developmental Medicine, Johannes Kepler University Linz, Linz, Austria; 3 Department of Pediatrics I, Medical University of Innsbruck, Innsbruck, Austria; 4 Department of Pediatrics, The Hospital for Sick Children, University of Toronto, Toronto, Ontario Canada; 5 Department of Pediatrics III, Medical University of Innsbruck, Innsbruck, Austria; 6 Department of Pediatric Nephrology, Hospital Memmingen, Memmingen, Germany; 7 Institute of Hygiene & Medical Microbiology, Medical University of Innsbruck, Innsbruck, Austria

**Keywords:** CFH-Ab, Hemolytic uremic syndrome, Immunosuppressive therapy

## Abstract

**Background:**

The complement factor H antibody (CFH-Ab)–associated hemolytic uremic syndrome (HUS) forms a distinct subgroup within the complement-mediated HUS disease spectrum. The autoimmune nature of this HUS subgroup implies the potential benefit of a targeted immunosuppressive therapy. Data on long-term outcome are scarce.

**Methods:**

This observational study evaluates the clinical outcome of 19 pediatric CFH-Ab HUS patients from disease onset until their 5-year follow-up.

**Results:**

All but one relapse occurred during the first 2 years, and patients who had no relapse within the first 6 months were relapse-free until the end of the observation period. Kidney function at disease onset determines long-term kidney function: all individuals with normal kidney function at disease onset had normal kidney function after 5 years, and all patients with reduced kidney function at onset had impaired kidney function at the last follow-up. Level of CFH-Ab titer at disease onset was not correlated with a higher risk of recurrences or worse long-term outcome after 5 years. Resolution of CFH-Ab titers after 5 years was common.

**Conclusions:**

CFH-Ab HUS patients have a varied overall long-term course. Early relapses are common, making close surveillance during the first years essential, regardless of the initial CFH-Ab titer.

## Introduction

Complement factor H antibody (CFH-Ab)–associated hemolytic uremic syndrome (HUS) forms a distinct subgroup of primarily complement-mediated thrombotic microangiopathies (TMA). CFH-Ab-associated HUS is reported in 6–25% of atypical HUS (aHUS) patients with pediatric onset of the disease [1–4]. Colleagues in India reported a much higher incidence (56%), which is so far not understood [5].

TMA is characterized by endothelial cell activation and secondary thrombus formation in the microvasculature, resulting in thrombocytopenia, hemolytic anemia, and organ failure. In some cases, endothelial cell activation is a result of complement deposition on the endothelial cell surface due to dysfunctional regulation. In CFH-Ab-associated HUS, complement dysregulation is caused by antibodies that decrease the function of the major complement regulator CFH [6].

It is generally accepted that early diagnosis of CFH-Ab HUS and its distinction from other TMA forms is highly important as the autoimmune nature of this TMA implies the potential benefit of targeted immunosuppressive (IS) therapy [6, 7]. Although no randomized controlled study on the efficacy of a therapy for CFH-Ab HUS is available, a consensus report [7] proposes the following approach for CFH-Ab HUS: therapy initiation with eculizumab or plasmapheresis (PP) within the first 2 days of HUS onset. Initiation of IS induction therapy should be based on the severity of extrarenal manifestations and followed by maintenance therapy with mycophenolate mofetil (MMF) and corticosteroids for at least 1 year. However, specifics on which immunosuppressive drug to use as well as the decision to use either eculizumab and/or PP remains an individual and empirical decision for each physician.

The aim of this observational study was to establish better knowledge on the long-term outcome of patients with pediatric onset CFH-Ab HUS.

## Methods

### Participants

Patient data were collected both prospectively and retrospectively by the Austrian-German-HUS-NET study group in cooperation with several participating centers (www.hus-online.at). Only CFH-Ab-positive patients with disease onset < 18 years were included in the study.

Informed consent was given by the patients or their guardians, and the study was performed according to the Declaration of Helsinki (2000), with approval of the local ethics board (Medical University of Innsbruck, Austria). The study includes data from 2002 to 2016. Data were collected from every patient at four times: initial diagnosis and 1, 2, and 5 years after diagnosis. Standardized questionnaires were used. Completed sets of questionnaires from 19 patients, serum samples from the acute phase and all follow-up examinations from 9 patients, and additional serum samples from all other included patients were analyzed.

All patients presented with the criteria for diagnosis of HUS: hemolytic anemia, thrombocytopenia, and kidney dysfunction. Kidney dysfunction was defined by serum creatinine levels greater than normal values according to age and/or urine protein-to-creatinine ratio > 0.2 g/g.

Relapse was defined by recurrence of hemolytic anemia and/or thrombocytopenia and/or kidney dysfunction at least 2 weeks after a patient has gone into either complete (no signs of hemolytic anemia or thrombocytopenia and normal kidney function) or partial remission (no signs of hemolytic anemia or thrombocytopenia but chronic kidney disease (CKD) and/or proteinuria).

### CFH-Ab assessment

CFH-Ab titers were determined using ELISA as previously published [1, 3]. However, due to the fact that disease onset in some patients was prior to 2005, the year when CFH antibodies were first described [4], initial titers are missing for some patients.

### Statistical analyses

Data were calculated using IBM-SPSS version 22 and Microsoft Excel. Comparison of metric variables was conducted by independent *t* test for normally and Mann-Whitney *U* test for non-normally distributed metric variables; Shapiro-Wilk test was used to test for normal distribution. Comparison of nominal variables was conducted by Fisher’s exact test. For the comparison of more than two groups, Kruskal-Wallis test was used for metric scale and Fisher’s exact test was used for nominal scale. Dependency was determined either by Pearson’s correlation coefficient (*r*) or by Spearman’s rho (*r*_*s*_). A significance level of 0.05 was applied.

## Results

### Patients show continued markers of TMA and kidney dysfunction beyond the acute phase

Data sets of 19 CFH-Ab HUS patients (8 female and 11 male) were analyzed for this study. Mean age at disease onset was 7.7 ± 2.7 years (range: 3.5–12.75). Table 1 lists clinical disease manifestations and the main laboratory data at disease onset and during 5-year follow-up. Elevated lactate dehydrogenase (LDH), low C3 levels, and kidney dysfunction continued to be abnormal beyond the acute phase.

**Table 1:** Clinical symptoms and laboratory findings

	Initial	1st year	2nd year	5th year
Clinical symptoms	19/19	17/19	16/19	16/18
Arterial hypertension	72% (13/18)	74% (14/19)	79% (15/19)	83% (15/18)
Hematuria	75% (6/8)	40% (6/15)	38% (5/13)	21% (3/14)
Proteinuria	75% (6/8)	78% (14/18)	73% (11/15)	44% (7/16)
Reduced kidney function	68% (13/19)	58% (11/19)	53% (10/19)	28% (5/18)
Abnormal kidney ultrasound	N/A	62% (5/8)	50% (4/8)	56% (5/9)
Abnormal ABPM	N/A	40% (2/5)	60% (3/5)	50% (2/4)
Extrarenal manifestation	58% (11/19)	21% (4/19)	5% (1/19)	11% (2/18)
Laboratory examinations				
Minimum hemoglobin, g/dL	5.7 ± 1.1	11.9 ± 2.4	12.3 ± 1.6	12.7 ± 1.8
	(5.0–6.6)	(10.8–13.7)	(11.4–13.2)	(12.3–13.8)
	(*N* = 18)	(*N* = 15)	(*N* = 18)	(*N* = 18)
Elevated LDH*	100% (17/17)	67% (6/9)	27% (3/11)	11% (1/9)
Decreased haptoglobin*	88% (7/8)	40% (2/5)	60% (3/5)	0% (0/4)
Minimum platelets, 10^9^/L	29 ± 12	265 ± 94	269 ± 65	278 ± 72
	(21–35)	(193–347)	(214–312)	(231–332)
	(*N* = 17)	(*N* = 14)	(*N* = 18)	(*N* = 18)
Maximum creatinine, μmol/L	390 ± 280	222 ± 311	217 ± 319	145 ± 236
	(196–500)	(67–188)	(54–94)	(56–78)
	(*N* = 17)	(*N* = 18)	(*N* = 19)	(*N* = 18)
eGFR, ml/min/1.73 m^2^	75 ± 56	82 ± 44	87 ± 45	105 ± 25
	(12–132)	(51–118)	(68–121)	(80–125)
	(*N* = 8)	(*N* = 13)	(*N* = 13)	(*N* = 11)^#^
CFH-Ab titer, AU/ml	1533 ± 481	604 ± 375	<100 (*N* = 2);	<100 (*N* = 10);
	(959–1933)	(368–809)	461 ± 224	290 ± 142
			(264–650)	(135–394)
	(*N* = 10)	(*N* = 14)	(*N* = 15)	(*N* = 8)
C3 level, mg/dL	76 ± 29	93 ± 27	87 ± 18	100 ± 17
	(56–95)	(67–110)	(69–101)	(82–118)
	(*N* = 16)	(*N* = 12)	(*N* = 12)	(*N* = 10)
Low C3 level*	65% (11/17)	67% (6/9)	33% (3/9)	13% (1/8)
C4 level, mg/dL	21 ± 9.7	26 ± 16	21 ± 7.5	23 ± 12
	(12–24)	(13–34)	(17–24)	(13–30)
	(*N* = 12)	(*N* = 11)	(*N* = 10)	(*N* = 9)
Low C4 level*	14% (2/14)	14% (1/7)	0% (0/6)	0% (0/6)

The number of evaluable answers is indicated in parentheses (*N*). Data is presented as absolute number of cases per number of evaluable patients (*n*/*N*) or as mean ± standard deviation (interquartile range)

*CNS* central nervous system, *LDH* lactate dehydrogenase, *eGFR* estimated glomerular filtration rate calculated with the Schwartz formula

*According to local references used by the respective laboratory, which was unavailable in several cases

^#^Excluding the two patients who underwent renal transplantation

Kidney function improved over time; mean eGFR was 82.3 ml/min/1.73 m^2^ at 1-year follow-up and 105.3 ml/min/1.73 m^2^ after 5 years. Two patients were excluded from this analysis as they underwent kidney transplantation [8, 9].

Interestingly, LDH continued to be elevated in 67% at 1-year follow-up, thereafter in 27% and 11% at 2- and 5-year follow-up, respectively. Decreased C3 levels were still found in 67% of the patients 1 year after diagnosis; this percentage decreased significantly thereafter. On the other hand, most of the patients (*n* = 11) showed normal platelet counts and hemoglobin levels above 10.0 g/dl at all times.

Mean CFH-Ab titer levels decreased over time and were considered normal in two patients at 2 years and ten patients (55%) at 5 years after disease onset.

### Extrarenal manifestations are common in CFH-Ab patients

Extrarenal manifestations at disease onset were documented for 10 patients (Table 2) and mainly involved gastrointestinal symptoms such as diarrhea and vomiting. At 1-year follow-up, four patients showed extrarenal involvement. At the 2-year follow-up, only one case of extrarenal complication was found (patient 19 still had dilated aorta); however, at the 5-year follow-up, extrarenal involvement was found in two patients (in patient 19 with the dilated aorta, and in patient 6, who had no previous extrarenal symptoms, left ventricular hypertrophy was detected in the setting of arterial hypertension). Patient 8 died during the third year after being diagnosed with non-compaction cardiomyopathy. He was on eculizumab therapy for about 1 year before his death. Except for age at disease onset (significantly higher in patients with extrarenal symptoms; *n* = 19, Spearman rank correlation coefficient = 0.558, *p* < 0.02), no further significant differences between patients with and without extrarenal complications were found.

**Table 2:**
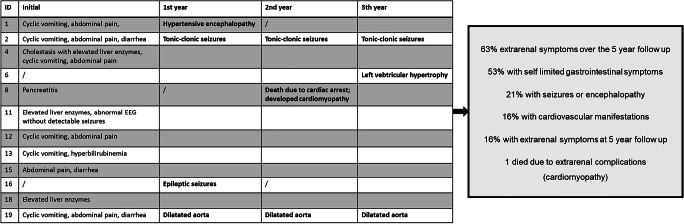
Extrarenal complications during the 5-year follow-up period

### Patients with CFH Ab HUS received a variety of treatments

Initially, 68% (13/19) of patients required kidney replacement therapy; however at 1- and 2-year follow-up, only 26% (*n* = 5) of the patients remained on dialysis (Table 3). After 5 years, two out of those five patients were transplanted, another two were still dependent on dialysis, and one patient was started on eculizumab and was able to stop dialysis.

**Table 3:** Therapy at disease onset and during follow-up

	Initial	1st year	2nd year	5th year
Kidney replacement therapy				
Dialysis	68% (13/19)	26% (5/19)	26% (5/19)	11% (2/18)
Transplanted	0% (0/19)	0% (0/19)	0% (0/19)	11% (2/18)
TMA-specific therapy				
Plasma therapy	95% (18/19)	53% (10/19)	26% (5/19)	6% (1/18)
Plasma infusion	16% (3/19)	21% (4/19)	11% (2/19)	0% (0/18)
Plasmapheresis	32% (6/19)	26% (5/19)	16% (3/19)	6% (1/18)
Both	47% (9/19)	5% (1/19)	0% (0/19)	0% (0/18)
Immunosuppressive/-modulatory therapy	16% (3/19)	42% (8/19)	37% (7/19)	44% (8/18)
Steroids	16% (3/19)	21% (4/19)	5% (1/19)	11% (2/18)
Mycophenolate mofetil	0% (0/19)	21% (4/19)	32% (6/19)	33% (6/18)
Rituximab	0% (0/19)	21% (4/19)	11% (2/19)	0% (0/18)
IV immunoglobulins	0% (0/19)	16% (3/19)	0% (0/19)	11% (2/18)
Biologicals				
Eculizumab	0% (0/19)	0% (0/19)	5% (1/19)	6% (1/18)
Therapy of sequelae				
Antihypertensive drugs	47% (9/19)	74% (14/19)	79% (15/19)	83% (15/18)
Diuretics	32% (6/19)	32% (6/19)	21% (4/19)	28% (5/18)
Antiepileptics	0% (0/19)	0% (0/19)	5% (1/19)	6% (1/18)
Other drugs	26% (5/19)	16% (3/19)	21% (4/19)	22% (4/18)
Packed RBC	84% (16/19)	0% (0/19)	0% (0/19)	0% (0/18)
Platelet transfusion	21% (4/19)	0% (0/19)	0% (0/19)	0% (0/18)
Antiplatelet drugs	5% (1/19)	0% (0/19)	0% (0/19)	0% (0/18)
Heparin	11% (2/19)	0% (0/19)	0% (0/19)	0% (0/18)
No therapy	0% (0/19)	16% (3/19)	21% (4/19)	17% (3/18)

Data is presented as absolute number of cases per number of evaluable patients (*n*/*N*)

**Fig. 1:**
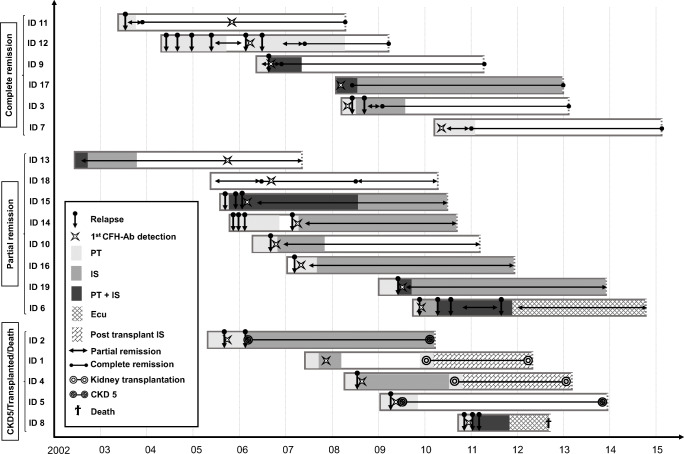
Swimmer plot grouped according to the outcome. The outcome after the 5-year follow-up period determines the group assignment. In patients 1, 3, 4, 5, 7, 11, 15, 16, 17, and 18, CFH-Ab titers dropped and are negative at 5-year follow-up. In patients 2, 6, 9, 10, 12, 13, 14, and 19, CFH-Ab titers are still detectable at 5-year follow-up but in the low titer range (> 100 and < 600 AU/ml). X-axis: years of observation. Beginning of the bars refers to onset of disease, which was quasi identical to the timepoint of HUS diagnosis (maximum of 2-week difference). Y-axis: group assignment and patient ID. Complete remission: normalization of LDH, haptoglobin, hemoglobin, and platelets in combination with normal kidney function. Partial remission: normalization of LDH, haptoglobin, hemoglobin, and platelets but presence of chronic kidney disease and/or proteinuria [13]. AU arbitrary units, CFH-Ab complement factor H antibodies, CKD stage 5 chronic kidney disease, Ecu eculizumab, HUS hemolytic uremic syndrome, IS any kind of immunosuppressive therapy except eculizumab, PT plasma therapy (any kind)

Except for one patient who developed stage 5 CKD directly after the first TMA episode (and who was transplanted thereafter [8]), all other patients received some kind of targeted therapy (plasma therapy (PT) and/or IS) at initial manifestation and 1-year follow-up (Table 3; Fig. 1). At 5-year follow-up, 9 out of 18 patients were still on CFH-Ab-targeted therapy (Fig. 1).

Eculizumab therapy was initiated in a second patient shortly after the 2-year follow-up and was still administered at the 5-year follow-up. Two years after diagnosis and prior to eculizumab, the patient continues to show signs of hemolysis, his CFH-Ab titer was slightly elevated at 420 AU/ml and was on chronic dialysis. At 5-year follow-up, the patient had regained sufficient kidney function (eGFR: 76 ml/min/1.73 m^2^) and was able to come off dialysis. Left ventricular hypertrophy was noted on echocardiography.

### Relapses are common within the first 6 months of disease onset

**Fig. 2:**
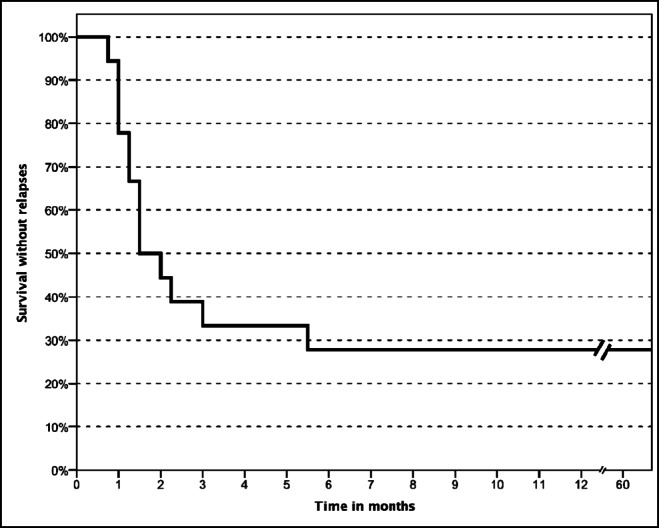
Relapse-free survival curve. As further detailed in the text, all of the first relapses occurred within the first 6 months after disease onset. One patient is missing as the exact timepoint of the first relapse is not known (but was before 6 months after disease onset)

Relapses were common, with fourteen patients (74%) suffering from at least one relapse (Table 4; Figs. 1 and 2). All patients had their first relapse during the first 6 months after diagnosis; the longest documented period from diagnosis to first relapse was 5.5 months. Only three patients had one or more relapses in the second year, and only one patient had a relapse in the third year (Table 4). The average time to the first relapse was 1.8 ± 1.3 months (range: 3 weeks–5.5 months); the average time to the last relapse was 7.8 ± 8.3 months (range: 1.8–28.0 months). On average, patients with relapses had 2.3 ± 1.6 relapses (range 1–6). Seven patients had only one relapse, 2 patients showed two relapses, 1 patient had 3 relapses, 3 patients showed 4 relapses, and one patient showed 6 relapses.

**Table 4:** Distribution of relapses over the follow-up period

	< 6 m	6 < 9 m	9 < 12 m	12 < 24 m	≥ 24 m
Patients with ≥ 1 relapse	*n* = 14	*n* = 2	*n* = 3	*n* = 3	*n* = 1
Relapses (% of all relapses)	21 (67.7)	2 (6.5)	3 (9.7)	4 (12.9)	1 (3.2)
Patients with > 1 relapse	*n* = 5	None	None	*n* = 1	None
Range of relapses	1–3	1	1	1–2	1

A significant correlation from timepoint of first relapse to further relapse probability was noted, but no relation to outcome parameters at 5-year follow-up was observed (Table 5). There was no significant difference in any of the clinical or laboratory parameters at disease onset between patients with relapses during the first 6 months (*n* = 14) and those without (*n* = 5; Table 4). Moreover, patients relapsing during the first 6 months did not show higher initial CFH-Ab titers.

**Table 5:** Statistical relations of patients with and without relapse within the first 6 months

	≥ 1 relapse within 6 months from disease onset	No relapse within 6 months from disease onset
≥ 1 relapse > 6 months from disease onset	12	0
No relapse >6 months from disease onset	2	5
Fisher’s exact	*p* < 0.002	
Complete remission at 5 years	4	2
Partial remission at 5 years	6	2
CKD5D/KT/Death at 5 years	4	1
Fisher’s exact	n.s.	

*CKD5D* chronic kidney disease stage 5 treated by dialysis, *KT* kidney transplant, *n.s.* not significant

**Fig. 3:**
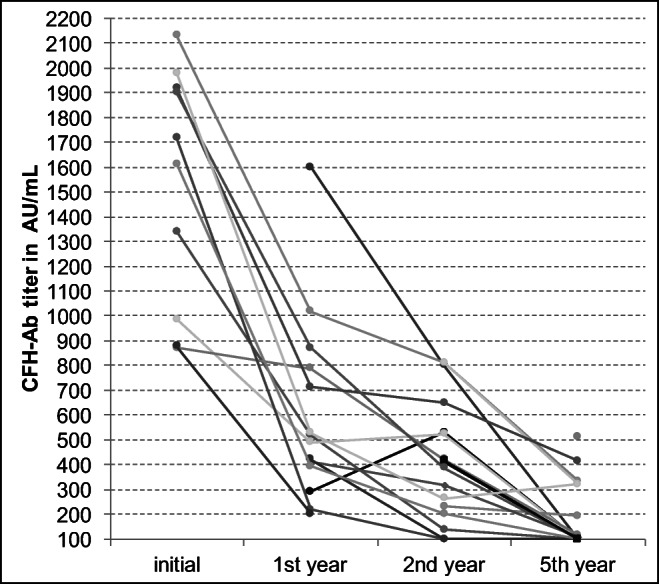
Evolution of CFH-Ab titers over 5-year follow-up period. Steady decrease of available CFH-Ab titers from disease onset up to 5-year follow-up (at 5 years, 55% of patients with CFH-Ab titers under cut-off of 100 AU/ml)

A steady decrease of available CFH-Ab titers from disease onset up to 5-year follow-up was observed (Fig. 3).

The available data on symptoms and therapy during relapses are incomplete. In all evaluable events (10 relapses of seven individual patients), patients presented with proteinuria and reduced kidney function during the relapse, and plasma therapy was first-line treatment.

### Three quarters of patients are in complete or partial remission after 5 years

Eighteen patients (95%) were alive at the 5-year follow-up (Fig. 1). Thirty-two percent (*n* = 6) were in complete remission, four of those showing arterial hypertension and two still with detectable CFH-Abs. Partial remission was observed in 42% (*n* = 8), including 7 with arterial hypertension and 5 with detectable CFH-Abs. Including the patient who died during the third year, 26% (*n* = 5) presented with CKD 5 (one of those with still detectable CFH-Abs) during follow-up.

Altogether, 5 years post initial manifestation, 68% (*n* = 13) showed a normal eGFR (two of those transplanted), eight of them with some signs of kidney damage in the form of proteinuria, hematuria, or pathological findings on kidney ultrasound.

All patients with impaired kidney function at the 5-year follow-up already presented with reduced kidney function at initial manifestation. Patients with normal kidney function at initial manifestation still had so after 5 years.

Fifteen patients had arterial hypertension at the 5-year follow-up. Of the 13 patients with high blood pressure at the initial manifestation, only one recovered and regained normal blood pressure after 2 years. Five patients developed arterial hypertension at any point in follow-up. An analysis of the patient’s initial characteristics, laboratory findings, and therapy in regard to their outcome after 5 years (i.e. complete remission, partial remission, CKD 5, or death) showed no statistically significant differences between the groups.

## Discussion

This study evaluates the clinical outcome of 19 CFH-Ab-positive pediatric HUS patients from disease onset over a 5-year follow-up period, making it the longest pediatric follow-up study of CFH-Ab-positive patients.

In general, relapses are common in CFH-Ab aHUS patients. Dragon-Durey et al. reported relapses in 25 of 44 CFH-Ab HUS patients, whereof 17 had their first relapse within 6 months from onset; however, in five patients, a relapse was seen after more than 12 months [10]. Sinha et al. reported disease relapse in only 14 of 122 patients and argued that this number is comparatively low because of the stringent induction of IS maintenance therapy in their patients [5]. Of the 19 patients described here, relapses were seen in 14 cases. The first relapse for each patient occurred within 6 months from diagnosis, and patients who did not suffer a relapse in the first 6 months were relapse-free until the end of the observation period. These findings emphasize the importance of treatment and close follow-up within the first year. Interestingly, in all sufficiently documented relapses (10 relapses of seven individual patients), proteinuria was described. Ardissino et al. suggested the implementation of urine test strips for home monitoring and early diagnosis of possible relapses in cases of discontinued eculizumab therapy [11]. Based on our data on CFH-Ab patients, screening for proteinuria or change in the severity of proteinuria may serve as a reasonable and economical method to identify relapses even with home-based monitoring.

Overall, there was no connection between higher titers and disease severity noticed. Initial CFH-Ab titer did not serve as a predictor for long-term outcome; however, during relapses, the CFH-Ab titer was higher than during remission, thus the individual patient’s titer development may serve as potential predictor for relapses.

The occurrence of extrarenal manifestations of TMA is frequent in aHUS patients [12]. In CFH-Ab HUS patients, central nervous system (CNS) involvement has been found in 14 to 41% of patients [5, 10, 13]. Pancreatitis was found in 23% [10], and elevated liver enzymes were reported in 50% [10] and 57% [5] of the cases. In this study, 63% of patients were reported with extrarenal involvement. Twenty-one percent had a CNS involvement, 16% had a cardiovascular involvement, and 58% had some form of gastrointestinal involvement. For all but one patient, multiple extrarenal manifestations were registered. Of note, all the CNS and gastrointestinal involvements occurred during the initial phase or the first year, whereas the cardiovascular involvements became evident after the first-year follow-up. This might have been influenced by continuous arterial hypertension resulting in cardiac changes like left ventricular hypertrophy.

Treatment modalities varied greatly between patients and were partly influenced by time of presentation as treatment recommendations evolved over time. Two of the patients were treated with eculizumab during the follow-up period: patient 6 was treated with a combination of PT and IS at the first- and second-year follow-up and received a combination of PT and eculizumab from the third year onwards.

The therapy of patient 8 was changed during the second year from a combination of PT, IS, and intravenous immunoglobulins to eculizumab. He died after almost exactly 1 year on this therapy due to non-compaction myocardiopathy. The efficacy of eculizumab on extrarenal manifestations is a controversial issue—some case reports on successful therapy of extrarenal involvements with eculizumab are available [14–18].

In the literature, the percentage of patients who develop CKD 5 or die ranges from 34 to 63% [2, 5, 10, 13, 19], partial remission ranges from 25 to 83% [3, 5, 10, 13, 20], and complete remission from 0 to 25% [3, 10, 13, 20]. In the present cohort, 26% developed CKD 5 or died, 42% had a partial remission, and 32% had a complete remission after 5 years. There was no difference in clinical presentation or CFH-Ab titers between patients with favorable or non-favorable outcome.

Impaired kidney function at one of the follow-up appointments was in all cases preceded by reduced kidney function at the initial manifestation. Patients with normal kidney function during the initial phase had normal function at the 5-year follow-up, suggesting that kidney damage mainly occurs at the initial manifestation. Similarly, Geerdink et al. reported arterial hypertension in 67% of their patients in the long term [3]. In a large prospective long-term follow-up study (German-Austrian HUS-NET) on patients with STEC HUS, it was noted that 18% developed proteinuria and/or arterial hypertension within the 1- and 5-year follow-up. The increasing rate of arterial hypertension over the years supports our recommendation for a close and lifelong monitoring of CFH-Ab HUS patients, especially as nearly no data exists on the evolution of the disease beyond adolescence and early adulthood.

In conclusion, CFH-Ab HUS patients show early relapses and long-term follow-up depends on initial manifestation. Antibody titers do not correlate well with outcome and disappeared in most of our patients within 5 years.

Our findings stress the importance of ongoing surveillance, especially at the initial phase and during the first year regardless of the initial CFH-Ab titers, owing to frequent relapses especially during the early period.

## Data Availability

The datasets analyzed during the current study are available from the corresponding author on reasonable request.
